# Subtrochanteric shortening osteotomy during cementless total hip arthroplasty in young patients with severe developmental dysplasia of the hip

**DOI:** 10.1186/s12891-017-1857-x

**Published:** 2017-11-25

**Authors:** Duan Wang, De-Hua Li, Qi Li, Hao-Yang Wang, Ze-Yu Luo, Yang Yang, Fu-Xing Pei, Zong-Ke Zhou

**Affiliations:** 1 Department of Orthopedics, West China Hospital/West China School of Medicine, Sichuan University, 37# Wuhou Guoxue road, Chengdu, 610041 People’s Republic of China; 2 Key Laboratory of Birth Defects and Related Diseases of Women and Children, Ministry of Education, Sichuan University, Chengdu, 610041 People’s Republic of China; 3 Department of Nursing, West China Second University Hospital/West China Women’s and Children’s Hospital, Chengdu, 610041 China; 4 State Key Laboratory of Oral Diseases, West China Hospital of Stomatology, Sichuan University, Chengdu, 610041 People’s Republic of China

## Abstract

**Background:**

This retrospective study was designed to determine complications, functional and radiographic results of transverse subtrochanteric osteotomy during cementless, modular total hip arthroplasty (THA) in a series of active patients younger than 45 years with Crowe Type-III or IV developmental dysplasia of the hip (DDH).

**Methods:**

We followed 49 patients (56 hips) with DDH who were treated with cementless THA, where the acetabular cup was positioned in the anatomic hip center and where a simultaneous transverse femoral osteotomy was performed. Complication rate evaluation and clinical outcomes were measured by validated clinical scores and radiographic evaluation were performed at a mean follow up of 10 years (range, 4.8–14.3 years).

**Results:**

The mean limb-length discrepancy was reduced from 4.2 cm to 1.1 cm (*P* < 0.01). The mean Harris hip score (HSS) significantly improved from 40.6 points to 87.4 points (*P* < 0.01). Similarly, severity of low back pain, modified MAP, HOOS, and SF-12 also showed significant improvement (*P* < 0.01). There were 3 cases of postoperative dislocation, 3 cases of transient nerve palsy, 2 cases of nonunion, and 4 cases of intraoperative fracture. At 10 years follow-up, the estimated survival rate with any component revision as end points was 92%.

**Conclusion:**

The cementless THA combined with transverse subtrochanteric osteotomy is a reliable technique with restoration of a more normal limb, satisfactory clinical outcomes, and mid-term survival of components.

## Background

Characterized by anatomical abnormalities, biomechanical alterations, femoral deformities and severe soft tissue contractures, severe developmental dysplasia of the hip (DDH) increases the complexity of performing total hip arthroplasty (THA) [1, 2]. In most patients with Crowe Type-III or IV DDH, the true acetabulum is the best position for supporting cup due to sufficient bone stock and good biomechanics [3]. Therefore, placement of the acetabular cup in the true acetabulum has proved to obtain durable clinical and favorable biomechanical results of THA in such patients [4–6]. However, the limb may be lengthened by more than 4 cm during restoration of the anatomical hip rotation center, which resulted in overstretching of neurovascular structures and difficulty in reducing the hip. In addition, overstretching of peri-articular structures, including muscles and tendons, may lead to the dysfunction of the abductor muscles, hip stiffness, and early loosening of the components [7].

To address these concerns during THA for severe dysplasia, femoral shortening is sometimes necessary and an effective in most cases to correct femoral malrotation, facilitate the reduction, and equalize limb lengths without tension forces and increased risk of neurological traction injury [5, 7, 8]. Shortening osteotomy at different anatomic levels, including greater trochanter, subtrochanteric region,, and distal femur, has been described previously. Subtrochanteric osteotomies can be performed with various techniques, such as transverse, oblique, Z-shaped, and chevron-shaped, each of which has their own advantages and disadvantages [2, 9–11]. The main disadvantage of the osteotomy is the risk of fracture and nonunion. The transverse type of bone cutting is a simpler and effective technique with similar rotational stability compared with other types of osteotomy in young patients [10]. Compared with a non-modular standard stem, a modular stem may be desirable to provide good fit and fill in the femoral canal and to obtain torsional stability at the osteotomy site [12]. In our center, we have performed cementless THA combined with transverse subtrochanteric osteotomy with use of modular stem in most patients with severe dysplasia [13].

Several prior reports have described short- to mid-term results of cementless THA combined femoral osteotomy shortening using modular stem in limited number of patients with severe dysplasia. However, these reports have included mixed groups of cemented and cementless femoral and acetabular components, different types of femoral osteotomy techniques, or even no femoral osteotomy in patients with large age span [9, 11, 14–16]. Moreover, many patients with severe DDH have complained not only of hip pain but also of low back pain and needed intensive treatment for such pain [17]. Lumbar spine and hip arthritis often coexist. Some studies demonstrated that THA could relieve the low back pain in patients with hip arthritis [18]. Nevertheless, no previous studies evaluated the low back pain after THA combined with femoral shorting osteotomy in active young patients who present severe hip dysplasia.

The purpose of the present study was to evaluated (1) mid-term clinical outcomes, (2) radiographic evaluation, (3) complications, and (4) mid-term survival of components in a group of active young patients with Crowe type-III and IV DDH who underwent cementless THA with transverse simultaneous subtrochanteric shortening osteotomy. We hypothesized that the cementless THA combined with transverse subtrochanteric shortening osteotomy is a reliable method in restoration of a more normal limb and satisfactory clinical outcomes, with mid-term survival of components for young patients with Crowe type-III and IV DDH.

## Methods

### Ethics statement

The study protocol was approved by the local institutional review board of West China Hospital, Sichuan University. Informed consent (including patients’ details, images, or videos) was obtained from all participants. All experiments were performed in accordance with relevant guidelines and regulations.

### Patients

We retrospectively reviewed 53 consecutive patients (62 hips) in whom cementless THA combined with transverse subtrochanteric shortening osteotomies was performed for Type-III or IV DDH according to Crowe classification from 2002 to 2012 in joint registry. One patient (1 hip) did not attend due to health issues unrelated to the THA, and one patient (2 hips) did not attend owing to no longer being interested. Two patients (3 hips) were lost to follow up after surgery and could not be contacted by phone or email at least 5 attempts. In the remaining 49 patients (56 hips), the hip dislocation was presented unilaterally in 42 patients and bilaterally in 7 patients. In bilateral high dislocations, 5 patients had Crowe type-IV, and 2 patients had Crowe type-IV dislocation of one hip and Type-III of the other. Therefore, 8 hips (14.3%) were Crowe Type III, while the other 48 hips (85.7%) were Crowe Type IV.

Severe pain unresponsive to non-operative management, pelvic obliquity, and functional impairment with limp in daily activities were the indication for surgery. There were 9 males and 40 females with mean age of 36.9 years (range, 19–45 years) at the time of index THA. Previous Schanz osteotomy was performed in two patients. Modular femoral stem (S-ROM^**®**^, DePuy Orthopadics, Marsaw IN.) with porous-coated acetabular component (PINNACLE^**®**^, POROCOAT^**®**^, DePuy Orthopadics, Marsaw IN.) was identical in all hips. The mean postoperative follow-up period was 10.2 years (range, 4.8–14.3 years). The pre-, peri- and post-operative data and surgical data were prospectively collected onto a predefined data collection form for patients with DDH. Table 1 showed the demographics of our study group.

**Table 1:** Demographics and clinical results

Indicator	Pre-op clinical features		Overall (Pre-op)	Overall (Post-op)	*P* Value^a^
	Crowe Type-III	Crowe Type-IV			
No. of hips	8	48	56	56	
Gender (No. of pts)					
Male	0	9	9	–	
Female	8	34	40	–	
Affected side (No. of hips)					
Left	3	21	24	–	
Right	5	17	22	–	
Bilateral	0	10	10	–	
Harris Hip Score					
Mean in points (range)	54.6 (48–59)	38.2 (23–57)	40.6 (23–59)	87.4 (77–98)	<0.01
Rating (no. of hips)					
Excellent (90–100 points)	0	0	0	15	
Good (80–89 points)	0	0	0	39	
Fair (70–79 points)	0	0	0	2	
Poor (<70)	8	48	56	0	
PMA					
Mean in points (range)	7.6 (6–9)	6.5 (4–9)	6.6 (4–12)	16 (14–18)	<0.01
Pain	2.5 (2–3)	2.1 (1–4)	2.2 (1–4)	5.5 (5–6)	<0.01
Motion	2.4 (2–3)	2.3 (1–3)	2.3 (1–3)	5.2 (5–6)	<0.01
Function	2.6 (2–3)	2.0 (1–3)	2.1 (1–3)	5.3 (4–6)	<0.01
Rating (no. of hips)					
Excellent (18 points)	0	0	0	6	
Good (15–18 points)	0	0	0	43	
Fair (12–15 points)	0	0	0	7	
Poor (<12 points)	8	48	56	0	
Limp (no. of Pts.)					
Severe	3	28	31	0	
Moderate	4	15	19	3	
Slight	1	5	6	11	
None	0	0	0	42	
Limb-length discrepancy					
Mean in mm (range)	2.8 (2.1–3.7)	4.4 (2.3–6.5)	4.2 (2.1–6.5)	1.1 (0.6–1.4)	<0.01
< 1 cm (no. of hips)	0	0	0	24	
1–2 cm (no. of hips)	0	0	0	32	
2–3 cm (no. of hips)	5	4	9	0	
3–4 cm (no. of hips)	3	13	16	0	
4–5 cm (no. of hips)	0	18	18	0	
5–6 cm (no. of hips)	0	10	10	0	
> 6 cm (no. of hips)	0	3	3	0	
HOOS subscale scores					
Symptoms	10.8 (8–12)	8.8 (4–12)	9.1 (4–12)	16.6 (14–19)	<0.01
Pain	20.6 (17–24)	15.9 (6–23)	16.6 (6–24)	36.5 (34–40)	<0.01
Activities of daily living	34.1 (32–37)	28.7 (19–36)	29.4 (19–37)	60.9 (57–67)	<0.01
Sports and recreation	7.4 (5–8)	5.5 (3–8)	5.8 (3–8)	12.8 (10–16)	<0.01
Quality of life	6.9 (5–8)	5 (3–8)	5.3 (3–8)	13.7 (12–16)	<0.01
Trendelenburg sign (no. of hips)					
Yes	6	47	53	1	
No	2	1	3	55	
SF-12					
PCS	15.5 (12–17)	10.9 (6–16)	11.6 (6–17)	22.7 (19–25)	<0.01
MCS	18.3 (15–20)	13.8 (9–19)	14.4 (9–20)	25.6 (22–29)	<0.01

Values are expressed as mean with range

*MAP* Merle d’Aubigne and Postel, *PCS* Physical component summary, *MCS* Mental component summary, *ROM* range of motion, *HOOS* Hip dysfunction and Osteoarthritis Outcome Score

^a^ *P* < 0.05 is significant

### Surgical planning and procedures

All operations were planned with transparencies and performed with use of cementless femoral and acetabular components. An acetabular template was placed at anatomical hip center, and a femoral template was correspondingly interiorized. The amount of femoral shortening was calculated to improve leg length discrepancy and lengthen the leg by no more than 3–4 cm to avoid nerve injury. Three-dimensional computed tomography (CT) scans was utilized to evaluate the acetabular bone stock.

**Fig. 1:**
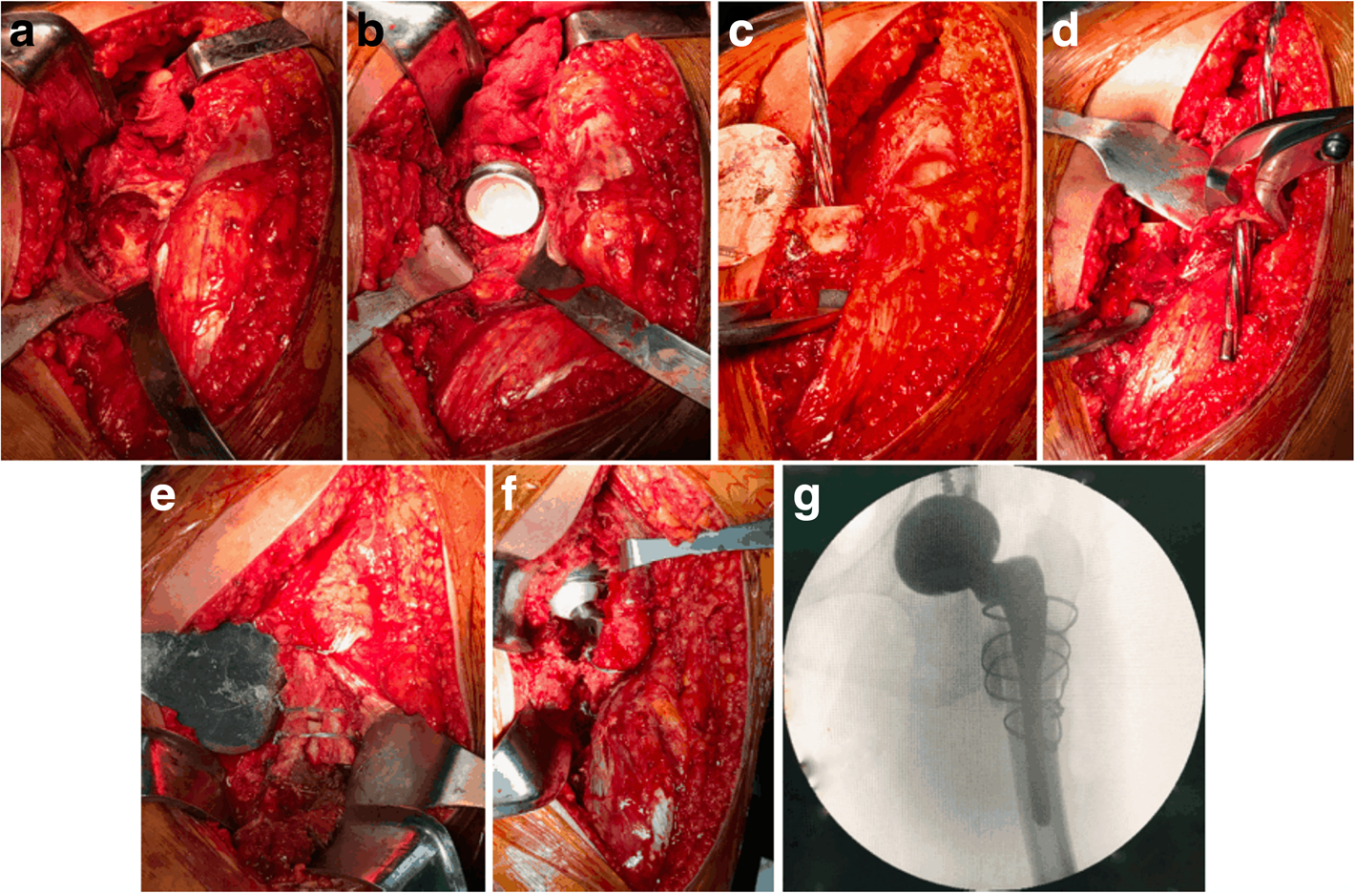
Intraoperative photographs of THA in a thirty-one-year-old woman with Crowe type-IV DDH. (**a**) Acetabulum was widened and deepened to obtain bleeding cancellous and femoral head autograft was applied. (**b**) The insertion of acetabular cup. (**c**) The straight axial intramedullary reaming process was conducted in the distal femur. (**d**) The proximal part of the femur was then prepared for the cementless implant. (**e**-**f**) The straight stem was then inserted into the femur across the osteotomy site, at which the gap was grafted with autogenous morselized bone and stabilized by cable fixation. (**g**) C-arm X ray was used to ensure the position of the cup and stem

All patients were operated on using a posterolateral approach in the lateral decubitus position. After resection of the femoral head and total capsulectomy were conducted, the position of the true acetabulum was reached and exposed through removal of fibrous scar tissue and osteophyte covering the true acetabulum. Soft-tissue release was applied in all hips. Transverse and round ligament was good indicator for recognizing true acetabulum. If the true acetabulum was not reachable due to the obstruction of the proximal part of the femur, a transverse femoral osteotomy approximately 1–2 cm distal to the lesser trochanter was performed. If there was proximal femoral canal deformity, an osteotomy was conducted at the level of the deformity. Elevation or split of a short section of the vastus lateralis was performed to approach the subtrochanteric area. Next, the proximal femoral fragment was translated anteriorly to reach the true acetabulum after the performance of transverse osteotomy. The acetabulum was widened and deepened with use of hemispherical reamers at a designated angle of antevertion and abduction to obtain bleeding cancellous, which indicated an interference fit between the anterior and posterior columns (Fig. 1a). As for insufficient coverage or severe acetabular bone defects, augmentation of the acetabulum was conducted by a structural autograft or titanium alloy (Ti-alloy) mesh combined with bulk bone grafting from the resected femoral head. Femoral head structural bone autografts fixed with screws were used in thirty-eight hips to improve coverage of the cup due to insufficient coverage, whereas Ti-alloy mesh and impaction bone grafting were used in nine hips owing to severe acetabular bone defects according to the three-dimensional CT. Implantation of the acetabular cup was conducted using press-fit technique (Fig. 1b). The median diameter of the acetabular cup was 48 mm (range, 44 to 52 mm). The 28-mm femoral head was used in twenty-six hips, 32-mm head in twenty-two hips, and 36-mm head in eight hips. Ceramic-on-ceramic wear bearing was applied in forty-eight hips, and metal-on-poly in eight hips.

After the acetabular component was inserted, attention was then directed to the more distal femur. A second transverse subtrochanteric osteotomy was conducted to shorten the femur by the planned amount. If proximal femoral canal deformity existed, correction of rotational deformity of the proximal femur was conducted before axial reaming through subtrochanteric osteotomy. Four cases (4 hips) with such femoral canal deformity were corrected in our series. The straight axial intramedullary reaming process was conducted in the distal femur, and the proximal part of the femur was then prepared for the cementless implant (Fig.1c-d). Then, sequential rasping was performed until the appropriate femoral component size was achieved. The proximal and distal bone fragments were then aligned, and they were properly rotated on the trial component. If a trial reduction of the hip could not be achieved following the first cut of the osteotomy, the additional osteotomy was gradually performed in sequence also with a transverse design to archive satisfactory hip reduction. The average amount of femoral shorting was 2.7 cm (range, 1.9–4.5 cm). After the proximal sleeve was implanted, the straight stem was then inserted into the femur across the osteotomy site when the rotational alignment of the 2 fragments was adjusted to allow approximately 15°-20°of anteversion of the femoral component (Fig. 1e). If necessary, prophylactic cable fixation could be used to prevent femoral fracture. After the insertion of femoral component, the gap at the osteotomy site was grafted with autogenous morselized bone and stabilized by cable fixation to enhance biologic healing in forty-six cases (Fig. 1f). In most cases, cortical autograft struts cut from the resected femoral bone fragment was applied for additional stabilization of the osteotomy site. After the insertion of femoral and acetabular components, C-arm X ray was used in most patients to ensure the position of the cup and stem (Fig. 1g).

### Postoperative rehabilitation

All patients were encouraged to conduct isometric exercises and active motion on a bed immediately after surgery. The patients walked with partial weight bearing under the protection of crutches for approximately 2 weeks. Then, gradually progressive full weight bearing was allowed 4–6 weeks depending on stability of the femoral stem and positively osseous healing at osteotomy site. The mean hospital stay was 7.2 days (range, 6–15 days).

### Clinical analysis

Clinical evaluation was conducted at 3 weeks, 6 weeks, 12 weeks, and 6 months after the surgery and annually thereafter until the last follow-up. A informative, concise follow-up data correction form was designed by the senior author (Z.K.Z.) for the patients with DDH [13]. The form included Hip dysfunction and Osteoarthritis Outcome Score (HOOS) (covering pain, symptoms, daily living, Sports, and quality of life), which is a validated and highly reproducible tool for evaluation of patient’s opinion about their hips, assessment of symptoms and functional limitations related to DDH, and description of their hip disability [19]. In addition, 12-item short-form health survey questionnaire (SF-12) is more sensitive than HOOS regarding the hip-related quality of life in younger active patients [13]. We decided to use HOOS and SF-12 as accurate measure of life quality and hip disability. In our center, the Harris Hip Score (HHS) and modified Merle d’Aubigne and Postel (MAP) hip score (including pain, motion, and gait function) are collected routinely to evaluate the function of the hip [13, 20, 21]. In addition to the validated patient-reported outcomes, all pre- and post-operative evaluations regarding the severity of limp and Trendelenburg test status were also achieved. The limb-length discrepancy (LLD) was also recorded in pre- and post-operative periods, which was measured from the anterior superior iliac spine to the medial malleolus suggesting the length discrepancy of lower extremities. At each visit, a specific questionnaire about the presence of low back pain and details of such pain was completed. We also asked the back-pain location and evaluated the intensity of the pain with use of the visual analogue scale (VAS). All complications were reviewed.

### Radiographic analysis

At each visit, all patients had standard anteroposterior and lateral radiographs of the hip, full-length view of the lower extremities. All radiographic measurements were adjusted for magnification. Radiographs were evaluated by authors who were not involved in the surgery. The amount of limb length was calculated by subtracting the amount of intraoperative femoral shortening from the amount of difference between the pre- and post-operative tip of the greater trochanter [22]. Bone union of the osteotomy site was evaluated on serial radiographs with the following radiographic criteria, including the presence of callus, cortical continuity, and no progressive gapping at the osteotomy site [23]. In addition, we also evaluated whether and when bone union occurred at the osteotomy site. Serial radiographs were also examined with respect to peri-prosthetic radiolucency, component migration, heterotopic ossification, osteolysis, and subsidence. Peri-prosthetic radiolucency was documented in acetabulum and femoral components according Delee and Charnley [24]. Heterotopic ossification was classified according to the method of Brooker et al. [25]. The seven zones around the femoral component on radiographs were described by Gruen et al. [26]. The mode of fixation of the femoral component was classified as bone ingrown, fibrous stable or unstable according to the system of Engh et al. [27]. Subsidence of femoral stem was also evaluated according to the method described by Engh et al. [27]. The loosening of acetabular cup was defined by the presence of progressive radiolucent lines >2 mm around the inserted cup, or migration, or a change in the position of the cup.

### Statistical analysis

Two-sided paired Student t test was used to analyze preoperative and postoperative continuous variables. Statistical significance was set at *P* < 0.05. The χ^2^ test was performed to analyze categorical variables. These data are presented as mean values with ranges. Kaplan-Meier was used to conduct analysis of cumulative survival rate of acetabular and femoral components. The end points for survival were defined as revision for any reason (including radiographic signs of loosening at the latest follow-up). Statistical analysis was performed with the use of SPSS Statistics software version 19.0 (IBM, Armonk, NY).

## Results

### Clinical outcomes

The mean HHS improved significantly from 40.6 points (range, 23–59 points) preoperatively to 87.4 points (range, 77–98 points) by the final follow-up (*P* < 0.01). Similarly, the mean modified MAP hip score improved from 6.6 points (range, 4 to 12 points) preoperatively to 16 points (range, 14 to 18 points) at the time of the latest follow-up, and the difference revealed significance (*P* < 0.01). For patient-reported outcomes, the mean HOOS and SF-12 scores significantly improved at the final follow-up (*P* < 0.01) (Table 1). Two of our patients were lost to follow-up.

Pre-operatively, the mean clinically measured LLD for unilateral THA was 4.2 cm (range, 2.1–6.5 cm) and it significantly improved to 1.1 cm (range, 0.6–1.4 cm) at the latest follow-up (*P* < 0.01), and no patients had >2 cm LLD postoperatively. At the final follow-up time, limp was moderate in three hips (5.4%), slight in eleven (19.6%), and none in 42 (75%) in the whole patient cohort. Moreover, positive Trendelenburg sign was presented in one patient (one hip) postoperatively. We did not include the subjects with opposite side DDH or other contralateral hip deformity.

**Fig. 2:**
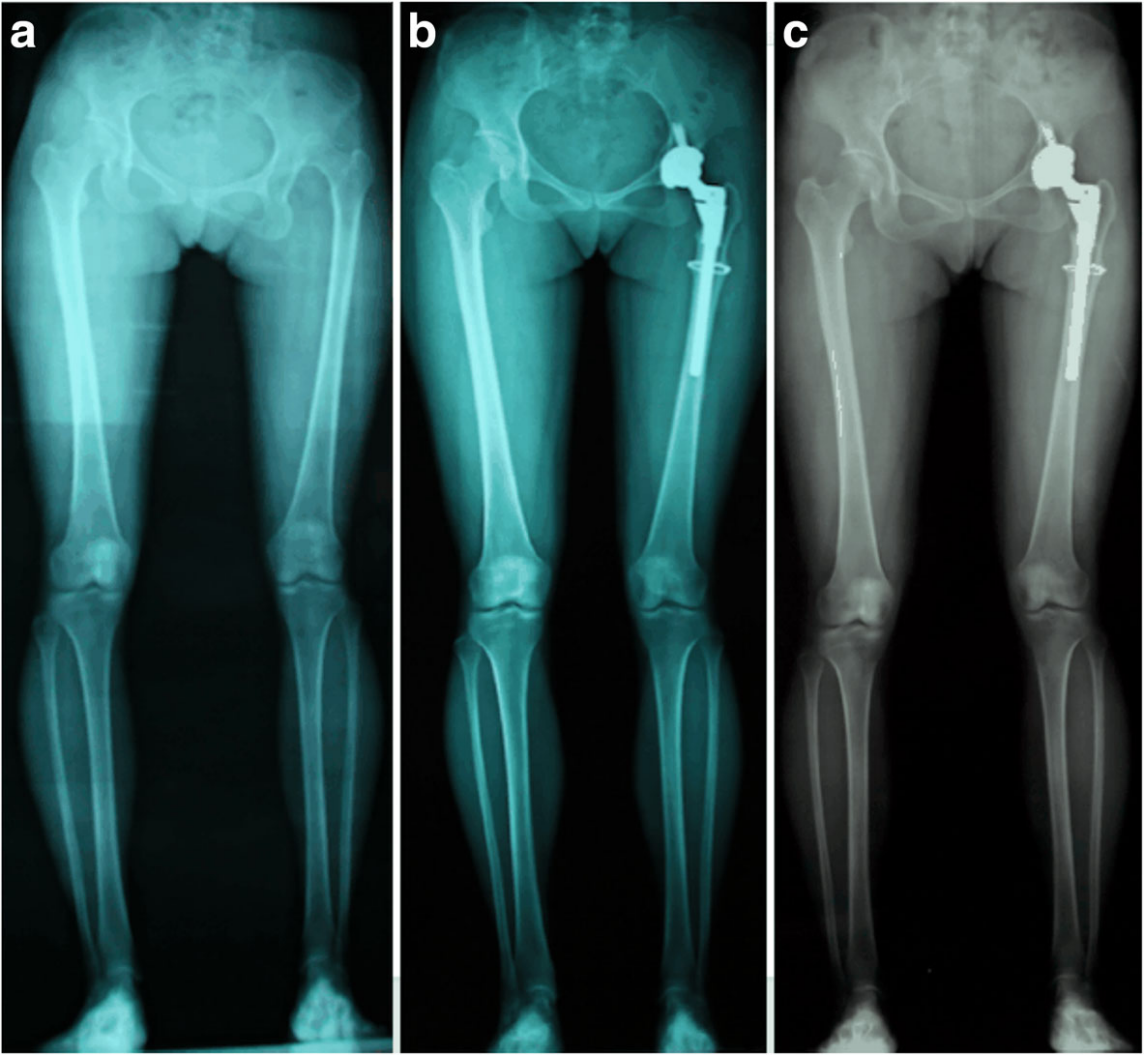
Full-length radiographs of a twenty-five-year-old woman with unilateral Crowe type-IV DDH. The serial post-operative radiographic image showed that pelvic inclination was normalized over time. (**a**) Pre-operative radiographic image; (**b**) Radiographic image after four years follow-up; (**c**) Radiographic image after nine years follow-up

The preoperative VAS score for low back pain was rated as severe in 10 patients (23.8%), moderate in 28 patients (66.7%), and mild in 4 patients (9.5%). At the final follow-up, the low back pain was moderate in 3 patients (7.1%), mild in 12 patients (28.6%), and none in 27 patients (64.3%). The low back pain improved over time, and the improvement was significant at every follow-up time point (*P* < 0.01) (Table 1, Fig. 2).

### Radiographic evaluation

**Fig. 3:**
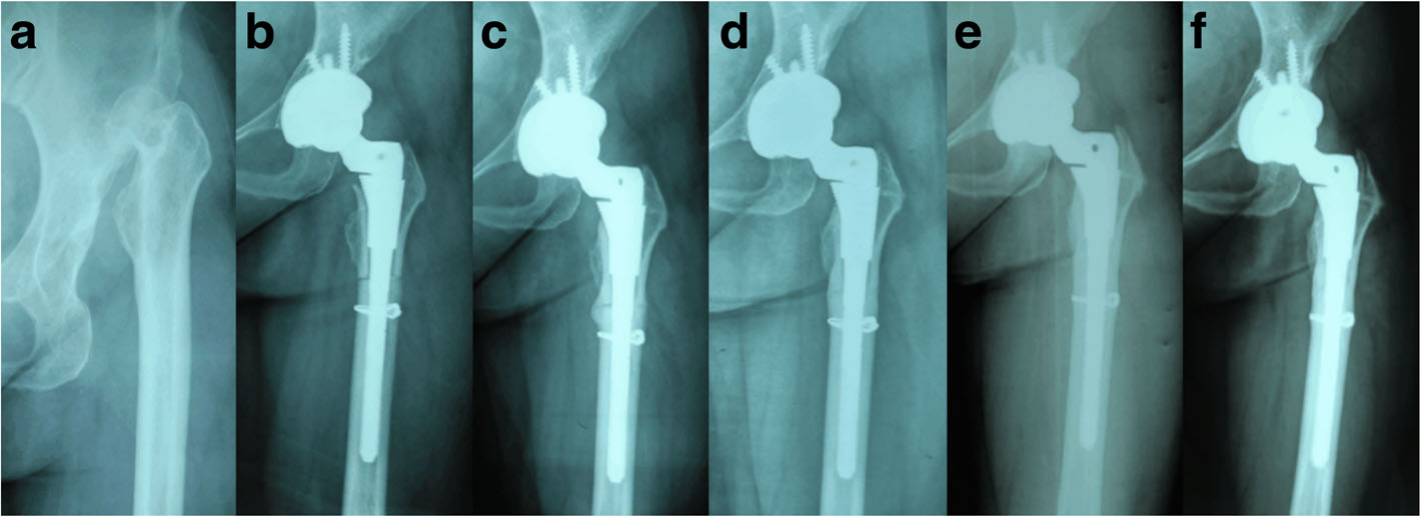
Radiographs of a twenty-nine-year-old woman with unilateral Crowe type-IV DDH. (**a**) Preoperative anteroposterior view. (**b**) Post-operative radiographic image at four months follow-up. Total hip arthroplasty was combined with simultaneous transverse osteotomy. Cerclage wires were placed at osteotomy site, and the acetabulum was reconstructed with screws. (**c**) Post-operative radiographic image at one year follow-up. (**d**) At four years follow-up, bone union was detected at the osteotomy site without radiolucent lines around the stem. (**e**) At eight years follow-up, the femoral and acetabular components showed no radiographic signs of loosening. (**f**) Post-operative radiographic image at eleven years follow-up. The femoral and acetabular components were stable

**Fig. 4:**
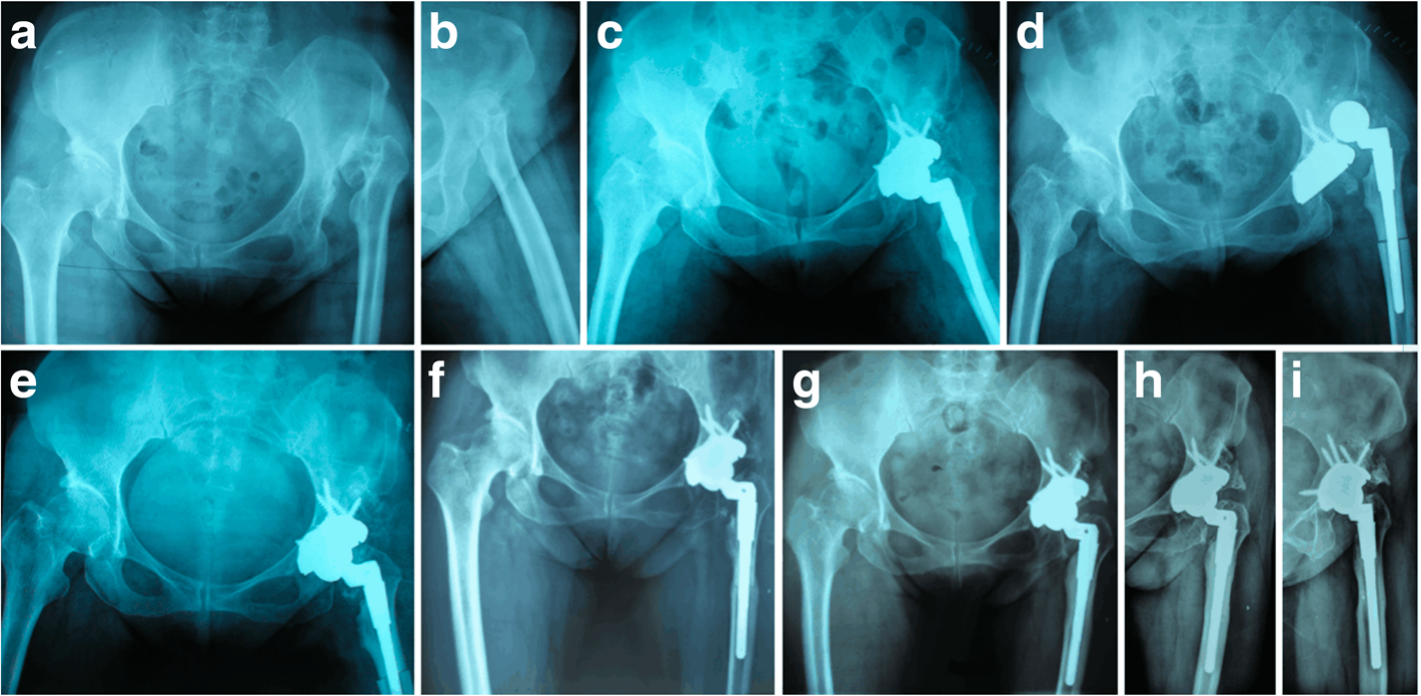
Radiographs of a forty-two-year-old woman with unilateral Crowe type-IV DDH. (**a**-**b**) Pre-operative anteroposterior view. (**c**) Radiographic image at postoperative 1 day. Total hip arthroplasty was performed combined with transverse osteotomy in left hip. (**d**-**e**) Postoperative anteroposterior view showed dislocation at 1-week follow-up, which was treated successfully through closed relocation. (**f**) Radiographic image after five years follow-up showed that no radiolucent lines around the femoral and acetabular components were identified. But osteolysis around the stem at the distal tip and heterotopic ossification were identified. (**g**-**i**) At eleven years follow-up, the postoperative Harris hip score was 97, and the femoral stem was judged to be stable with bone ingrowth

All acetabular components were placed in the position of true acetabulum (Fig. 3). Bone union of osteotomy site was archived in fifty-four hips with no complication, and the mean time to bone union was 6 months (range, 4–9 months). Serial radiographs showed two cases (4.1%) of nonunion at osteotomy site. One patient was treated successfully with bone grafting and medication, and showed bone union of the osteotomy site within 8 months without any complication at last follow-up time; the other one showed progressive stem subsidence and then the signs of radiographic loosening through the postoperative period, and it was revised with fully porous-coated stem at postoperatively 3.4 years and the new femoral component was stable at last follow-up time. Femoral stem that remained in situ after THA on radiographs indicated bone ingrowth in fifty hips (89.3%) at final follow-up time. Radiographic analyses of the femoral stem revealed radiolucency in three patients (6.1%) (three hips). However, no signs of progressive subsidence or loosening of femoral component were observed at the last follow-up in any hip. One patient (2.0%) (one hip) suffered from peri-prosthetic fracture around the femoral stem at 4.2 years, which was stabilized with a longer stem and cerclage wires, and fully healed without further complication. One patient (2.0%) (one hip) sustained peri-prosthetic fracture of distal femur at 5 years, and cerclage wires and bone graft were applied successfully for fixation. In addition, two patients (4.1%) (two hips) sustained acetabular fracture and loosening of the acetabular cup at postoperative five and seven years, respectively. They have been successfully treated by cup revision and bone grafting with no further complication (Table 2). Asymptomatic Brooker class-I and II heterotopic ossification were observed in three and two hips, respectively (Fig. 4). In addition, focal osteolysis was observed in five hips (Zone I in one hip, Zone II in two hips, and Zone 3 in two hips) on follow-up radiographs, but the cups showed bony incorporation and no surgery was required (Fig. 4).

**Table 2:** Complications

Case	Complication	Treatment	Results
5	Dislocation at 7 days	Closed reduction	No recurred dislocation
13	Dislocation at 2 years	Open reduction	No recurred dislocation
16	Dislocation at 2 weeks	Closed reduction	No recurred dislocation
9	Intraoperative femoral proximal fracture	Cerclage wires for fixation	No further complications
12	Intraoperative femoral proximal fracture	Cerclage wires for fixation	No further complications
19	Intraoperative femoral distal fracture	Cerclage wires for fixation	No further complications
24	Intraoperative femoral distal fracture	Cerclage wires for fixation	No further complications
14	Transient nerve injury	Medication and physical therapy	Full recovery
15	Transient nerve injury	Medication and physical therapy	Full recovery
21	Transient nerve injury	Medication and physical therapy	Full recovery
20	Nonunion of the osteotomy site, aseptic loosening of femoral component	Fully coated femoral component used in revision, bone graft at 3.4 years	Bone ingrowth and stable
35	Nonunion of the osteotomy site	Bone grafting and medication	Satisfactory clinical results
36	Peri-prosthetic fracture around the femoral stem	A longer stem and cerclage wires used in revision at 4.2 years	Bone ingrowth and stable
40	Peri-prosthetic fracture of distal femur	Cerclage wires and bone graft used in surgery at 5 years	Full healed
31	Acetabular fracture and thereby loosening of the acetabular cup	Cup revision and bone grafting at 5 years	No further complication
17	Acetabular and greater trochanter fracture, and screw breakage, and thereby loosening of the acetabular cup	Cup revision and bone grafting at 7 years	No further complication

### Complications

According to the Clavien-Dindo classification, the classification of surgical complications was grade I in 33 patients (67.3%), grade II in 9 patients (18.4%), and grade III in 7 patients (14.3%), respectively [28]. At the beginning of the series, an intraoperative nondisplaced fracture occurred in proximal femur in two hips and in distal femur in two hips, which were successfully treated with internal fixation with cerclage bands without further complication. Three patients (6.1%) (three hips) experienced transient nerve injury with weaknesses of ankle extension and dropfoot, who were treated with medication and physical therapy and fully recovered within 1 year without functional defects. Three patients (6.1%) (three hips) had dislocation postoperatively. In two cases, dislocation was treated successfully with closed reduction without further sequelae (Fig. 4). In one case, dislocation recurred two years after indexed THA and could not be resolved with closed reduction, and required open reduction. Moreover, four patients (8.2%) complained of longer or shorter contralateral limb length. But no limbs were longer or shorter based on the result of radiographic measurement. No vascular complications, infection and deep venous thrombosis was recorded (Table 2). In summary, three (6.1%) and four patients (8.2%) required reoperation and revision surgery in the follow-up period, respectively.

### Survivorship analysis

**Fig. 5:**
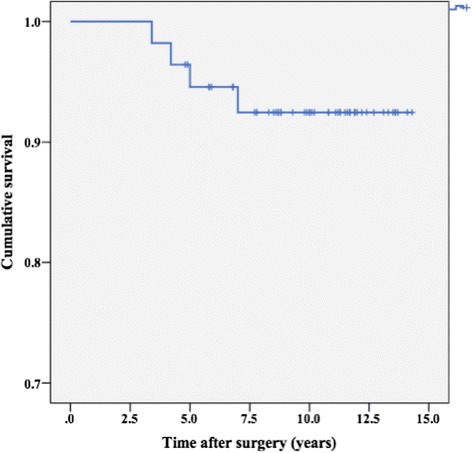
Kaplan-Meier survivorship curve with revision for any component as the end points

Kaplan-Meier survivorship with an end point of any component revision was 92% (95% confidence interval 84–99) at postoperative 10 years. Kaplan-Meier survivorship with an end point of aseptic loosening of the stem was 96% (95% confidence interval 87–99) at 10 years (Fig. 5).

## Discussion

Crowe Type-III or IV developmental dysplasia is technically demanding for cementless THA in young patients, such as anatomical and biomechanical abnormalities, soft tissue contractures, and femoral deformities. In THA for severe DDH, satisfactory clinical and radiographic results have been reported with placement of the acetabular component in the true acetabulum and a femoral shortening osteotomy. However, the majority of studies reported series of a small number of patients who treated with THA combined with a subtrochanteric shortening osteotomy have included different surgical techniques with mixed groups of cemented and cementless implants or combinations of other osteotomy techniques, and the results are not consistent [5, 6, 8, 23, 29–39] (Table 3). To our knowledge, this is the largest reported series of young patients with Crowe Type-III and IV DDH, in which the clinical and radiographic outcomes and related complications of exclusively THA combined with simultaneous transverse femoral subtrochanteric shortening osteotomy were evaluated. The major finding of this study was that the cementless THA combined with transverse subtrochanteric shortening osteotomy could improve function, resort a more normal limb, and provide stable fixation in hips with Crowe Type-III and IV DDH.

**Table 3:** Overview of relevant literature in the treatment of Crowe III or IV dysplasia combined with subtrochanteric femoral shortening osteotomy

Study	Year	Hips (n)	DDH type	Femoral osteotomy	Mean age (Yrs)	Stem Type	Acetabular Type	Clinical results		Revision	Nonunion	Aseptic Loosening		Introp fracture		Heterotopic ossification	Nerve injury	Dislocation	Survival (end points)	FU (Yrs)
								Pre-operation	Post-operation			Act AL	Fem AL	Introp AF	Introp FF					
Ozan et al.	2016	32 (25)	III&IV	Transverse	51 (35–70)	Cementless	Cementless	HHS: 49 (25–72)	HHS: 87 (74–94)	2 (6.2%)	0 (0%)	2 (6.2%)	0 (0%)	0 (0%)	0 (0%)	0 (0%)	0 (0%)	3 (9.3%)	93% (Loosening)	5.1
Verettas et al.	2015	66 (62)	III&IV	Transverse	46 (24–74)	Cementless	Cementless	NA	NA	4 (6%)	0 (0%)	0 (0%)	0 (0%)	0 (0%)	0 (0%)	0 (0%)	1 (1.5%)	2 (3%)	94% (Revision)	9
Sofu et al.	2015	73 (68)	III&IV	Transverse	47 (31–69)	Cementless (Stryker)	Cementless (Stryker)	HHS: 38.6 ± 2.9 Pain: 16 ± 3.7	HHS: 83.7 ± 6.8 Pain: 40 ± 4.6	6 (8.2%)	4 (5.5%)	0 (0%)	4 (5.5%)	0 (0%)	0 (0%)	2 (2.7%)	0 (0%)	1 (1.3%)	87% (Femoral loosening; nonunion)	5
Desteli et al.	2015	60 (52)	III&IV	Transverse	51 ± 13	Cementless (HA)	Cementless (HA)	HHS: 39(25–60) Pain: 3 (1–5) Function: 3(1–5) Motion: 2 (1–4)	HHS:93(80–100) Pain: 5.4 (3–6) Function: 4(3–5) Motion: 5 (4–6)	0 (0%)	0 (0%)	0 (0%)	0 (0%)	0 (0%)	0 (0%)	0 (0%)	0 (0%)	0 (0%)	NA	6.5
Ogawa et al.	2011	6 (6)	III&IV	Transverse	58 ± 7.2	Cementless (S-ROM)	Cementless (Stryker; Depuy)	JOA: 35.5 ± 5.7 Pain: 7.0 ± 3.4 Function:9.3 ± 1.3 Motion:11.2 ± 1.3	JOA: 72.3 ± 2.1 Pain: 35.8 ± 1.5 Function:14 ± 0.8 Motion: 16 ± 0.5	0 (0%)	0 (0%)	0 (0%)	0 (0%)	0 (0%)	0 (0%)	0 (0%)	0 (0%)	2 (33.3%)	100% (Loosening)	8.1
Akiyama et al.	2011	15 (11)	III&IV	Transverse	59 (42–77)	Cemented	Cemented	MAP: 8.1 ± 2.5	MAP: 15.1 ± 1.3	3 (20%)	3 (20%)	0 (0%)	0 (0%)	0 (0%)	0 (0%)	0 (0%)	0 (0%)	2 (13.3%)	80% (Revision)	3–10
Togrul et al.	2010	21 (14)	III&IV	Transverse	42 (33–52)	Cementless	Cementless	Pain: 2.9 (1–5) Motion: 4.4(3–5) Walking:3.7(2–5)	Pain: 5.2 (4–6) Motion:5.4(5–6) Walking:5.5(5–6)	1 (4 .8%)	0 (0%)	0 (0%)	0 (0%)	0 (0%)	0 (0%)	0 (0%)	0 (0%)	2 (9.5%)	95% (Revision)	3.4
Howie et al.	2010	35 (28)	III&IV	Transverse	47 (26–75)	Cemented	Cemented	OHS: 40.2 SF12: 79.6	OHS: 26.7 SF12: 103.3	7 (20%)	2 (3%)	2 (3%)	0 (0%)	0 (0%)	0 (0%)	0 (0%)	2 (6%)	3 (9%)	80% (Revision)	5.6
Biant et al.	2009	28 (22)	III&IV	Transverse	45 (23–74)	Cementless (S-ROM)	Cementless	HHS: 37 (19–69) SF12: NA WOMAC: 27	HHS:83(44–100) SF12: 95.6 WOMAC: 23	4 (14%)	0 (0%)	2 (7.1%)	0 (0%)	0 (0%)	2 (7.1%)	0 (0%)	1 (3.6%)	0 (0%)	NA	10
Park et al.	2007	24 (23)	III&IV	Transverse	49 (20–66)	Cementless	Cementless	HHS: 35.6	HHS: 81.7	4 (17%)	3 (12%)	1 (4%)	3 (13%)	0 (0%)	3 (13%)	0 (0%)	0 (0%)	1 (4%)	83% (Revision)	4.7
Gotze et al	2007	7 (7)	III&IV	Transverse	42 (29–64)	Cementless	Cementless	HHS: 43 (29–61)	HHS: 77 (66–90)	1 (14%)	0 (0%)	0 (0%)	1 (14%)	0 (0%)	0 (0%)	0 (0%)	0 (0%)	0 (0%)	86% (Revision)	1.5
Onodera et al.	2006	14 (13)	III&IV	Transverse	55 (44–69)	Cementless	Cementless	HHS: 38 (15–55)	HHS: 82 (35–93)	1 (7%)	1 (7%)	0 (0%)	1 (7%)	0 (0%)	6 (43%)	0 (0%)	NA	1 (7%)	93% (Revision)	5
Erdemli et al.	2005	25 (22)	III&IV	Transverse	44 (28–61)	Cementless	Cementless	HHS: 37 Pain: 2.3 Function: 2.3 Motion: 2.3	HHS: 95 Pain: 5.7 Function: 4.5 Motion: 4.4	4 (16%)	1 (4%)	0 (0%)	2 (8%)	1 (4%)	2 (8%)	1 (4%)	1 (4%)	1 (4%)	84% (Revision)	6
Masonis et al...	2003	11	III&IV	Transverse	49 (21–70)	Cementless	Cementless	HHS: 33 (22–45)	HHS:74(42–100)	5 (24%)	1 (9%)	0 (0%)	0 (0%)	0 (0%)	0 (0%)	0 (0%)	0 (0%)	0 (0%)	76% (Revision)	5.8
		10	III&IV	Transverse	49 (21–70)	Cemented	Cemented				1 (10%)	0 (0%)	0 (0%)	0 (0%)	0 (0%)	0 (0%)	0 (0%)	3 (30%)		5.8
Bruce et al.	2000	9	III&IV	Transverse	53 (26–77)	Cementless (S-ROM)	Cementless	HHS: 31 (20–25)	HHS: 81 (60–98)	2 (22%)	0 (0%)	0 (0%)	1 (11%)	0 (0%)	2 (22%)	0 (0%)	0 (0%)	1 (11%)	78% (Revision)	4.7
Present study	2016	56 (49)	III&IV	Transverse	36 (19–45)	Cementless (S-ROM)	Cementless (S-ROM)	HHS: 40 (23–59) Pain: 2.2 (1–4) Function: 2 (1–3) Motion: 2.3(1–3) PCS: 11.6 (6–17) MCS: 14.4(9–20)	HHS: 87 (77–98) Pain: 5.5 (5–6) Function: 5 (4–6) Motion: 5.2(5–6) PCS: 22 (19–25) MCS: 25 (22–29)	1 (1.8%)	1 (1.8%)	1 (1.8%)	0 (0%)	0 (0%)	3 (5.4%)	2 (3.6%)	3 (5.4%)	3 (5.4%)	98% (Revision)	10.1

*LLD* length discrepancy, *Intraop AF* intraoperative acetabular fracture, *Intraop FF* intraoperative femoral fracture, *Acet AL* acetabulum aseptic loosening, *Fem AL* femoral component aseptic loosening, *PW* polyethylene wear, *FNI* femoral nerve injury, *SNI* sciatic nerve injury, *OHS* oxford hip scores, *DVT* deep vein thrombosis, *PE* pulmonary embolism, *HHS* harris Hip Score, *MAP* Merle d’Aubigne and Postel, *JOA* Japanese Orthopaedic Association hip score

We noted some limitations of the current study. First, the number of patients was relatively small. Second, there was a retrospective study with no control group. Third, there was no evaluation of inter-observer reliability, and all operations were performed by five senior surgeons. Forth, low back pain was measured with VAS scale and lacks objective evidence in this study.

Previous studies reported that superior placement of the acetabular component could result in improper components orientation, unfavorable biomechanical environment and even higher rates of components loosening, especially in young patients with severe DDH [40, 41]. Therefore, we placed all acetabular cups in true acetabulum and restored the anatomical center of hip rotation in our series. In addition, considering the young age of our series and the risk of the need for potential revision surgery, structural acetabular autograft was conducted in most cases. Linde et al. found that the rate of mechanical loosening of the acetabular cup was 13% in the group with the anatomical position of acetabular components compared with 42% in the group with superior placement of the acetabular cups at 15 years follow-up, and the difference revealed significance [40]. In our series, only two patients (two hips) showed acetabular component loosening (3.5%) due to acetabular fracture and screw breakage.

Femoral shortening osteotomy is sometimes necessary to assist anatomic placement of acetabular cup without sciatic nerve compromise and excessive soft tissue tension [6, 31]. Subtrochanteric osteotomies can be performed at different anatomic levels with various surgical techniques, including Z-shaped, transverse, oblique, and double Chevron [2, 10, 11]. A biomechanical study by Muratli et al. compared four different subtrochanteric osteotomies techniques, and demonstrated that there was no single inherent feature increasing the stability of the osteotomy designs [10]. Li et al. performed a meta-analysis comparing transverse and modified osteotomy regarding complications and survivorship and no difference was found [42]. And, transverse osteotomy may be recommended compared with other techniques due to technical simplicity, convenience of adjusting the anteversion angle, correction of the rotational deformity, preservation of the proximal femoral metaphysis, relatively short learning curve for precise performance and minimal damage of the periosteum in patients with severe DDH [5, 42]. Therefore, transverse subtrochanteric osteotomy was utilized in all hips. Common complications in femoral shortening osteotomy were intraoperative femoral fracture with rates from 5 to 24% [6, 8, 23] and nonunion at the osteotomy site with rates from 7 to 22% [31, 32, 35]. Great attention should be paid to narrow and straight femoral canals, which are so vulnerable to fracture during broaching or insertion of femoral stem. Our union rate of osteotomy site was 96.4%. In our series, several methods were available to prevent nonunion. First, the interface between the osteotomy fragments should be cut as smoothly as possible. Second, autologous morselized bone grafts were used to augment the osteotomy gap, and the resected bone from femoral head was wrapped around osteotomy site. Third, great care was taken to minimize the damage of periosteum circumferentially during preparation of osteotomy sites and prophylactic cerclage wiring to keep the osteoblastic activity of periosteum. At the beginning of our series, no precautions were taken to decrease the risk of intraoperative femoral fracture, three cases of fracture were recorded that was successfully treated with cerclage bands and healed with no further complications. To obtain a strong fixation and minimize the risk of fracture, we subsequently paid more attention to fracture signs (e.g. sudden much more subsidence of the implant, rotational instability of stem at final insertion) during broaching and performed prophylactic cable fixation before the insertion of implant. The risk of intraoperative fracture decreased after prevention of fracture.

In this study, we consider dislocation as an important complication, which may compromise the press-fit between femoral component and femoral fragment and result in disengagement and early loosening of stem [43]. Total dislocation rates were 5.3% in our series that compared favorably with dislocation rates in other studies [8, 34, 36, 38]. The relatively low incidence of dislocation can be interpreted by the stronger muscle strength in younger and more active patients, proper cup orientation, and restoration of abductor muscles lever arm and center of hip rotation [3, 6, 7, 31]. Another important complication was transient nerve injury in three patients, which were recovered with no weakness or numbness in the limb by the latest follow-up evaluation. Some researchers reported that femoral lengthening increase the incidence of nerve palsy, and thereby they recommended lengthening the femur by <3 cm, < 4 cm, or <5 cm to avoid this complication [3, 12]. The transient nerve injuries occurred in our series due to relatively large amount of leg lengthening and less experience, and time-consumption at the beginning. We estimated the bone shortening based on preoperative templating, and believed that the leg lengthening of <3.5 cm may decrease the risk of nerve injury [13].

The function outcomes, including HSS, HOOS, and SF-12, improved significantly in our study group comparable to the results reported in the literature, which may be attributed to the relief of preoperative pain, improvement of LLD, and restoration of abductor muscles lever arm and femoral offset [5, 34, 35, 38]. In addition, we should pay more attention to chronic complications of the lumber spine and concomitant back pain, which was due to the compensation of lumber scoliosis and pelvic inclination for LLD. The results showed the significant improvement of patient’s low back pain in this study, which can be explained by the two reasons: (1) LLD was decreased, and (2) thereby pelvic inclination and excess scoliosis or lordosis was normalized over time after this procedure.

It is generally accepted that the rates of implant failure were higher in young and more active patients due to a high grade of physical activity and functional demand, compared with elderly and inactive patients. As a result of high failure rate of components in cemented THA, cementless THA with ceramic on ceramic can provide long-term stable fixation and became the gold standard in younger cases. In addition, modular standard stem is very suitable in cases with severe DDH with a need for femoral osteotomy, which provides good fit and fill in dysplastic narrow femoral canal and stable fixation of the metaphyseal and diaphyseal fragments, and thereby obtains torsional stability [12, 31]. With regards to the long-term survival, overall failure rate was 7.1% in our series, which was is comparable to other published studies recording a failure rate of 0–10% [31, 44].

## Conclusion

For young patients with Crowe Type-III or IV DDH, THA is very difficult due to the abnormal anatomy of the proximal femur and acetabular structure. The mid-term clinical and radiographic results of THA for these patients were good in our study. Thus, we demonstrated that cementless THA combined transverse subtrochanteric femoral shortening osteotomy in hips with Crowe Type-III or IV DDH is a reliable technique, which provides satisfactory outcomes, including acceptable rate of major lengthening-related complications, stable fixation of cementless implant, and significant improvement of function and relief of back pain.

## Abbreviations

DDH: developmental dysplasia of the hip
THA: Total hip arthroplasty
HOOS: Hip dysfunction and Osteoarthritis Outcome Score
HHS: Harris Hip Score
SF-12: 12-item short-form health survey questionnaire
MAP: Merle d’Aubigne and Postel
LLD: Limb-length discrepancy
VAS: Visual analogue scale
